# A Comparative Study on the Efficacy of NLRP3 Inflammasome Signaling Inhibitors in a Pre-clinical Model of Bowel Inflammation

**DOI:** 10.3389/fphar.2018.01405

**Published:** 2018-12-03

**Authors:** Carolina Pellegrini, Matteo Fornai, Rocchina Colucci, Laura Benvenuti, Vanessa D’Antongiovanni, Gianfranco Natale, Federica Fulceri, Marta Giorgis, Elisabetta Marini, Simone Gastaldi, Massimo Bertinaria, Corrado Blandizzi, Luca Antonioli

**Affiliations:** ^1^Department of Pharmacy, University of Pisa, Pisa, Italy; ^2^Department of Clinical and Experimental Medicine, University of Pisa, Pisa, Italy; ^3^Department of Pharmaceutical and Pharmacological Sciences, University of Padova, Padua, Italy; ^4^Department of Translational Research and New Technologies in Medicine and Surgery, University of Pisa, Pisa, Italy; ^5^Department of Drug Science and Technology, University of Turin, Turin, Italy

**Keywords:** anakinra, caspase-1, colitis, colon, NLRP3 inflammasome, interleukin-1beta, bowel inflammation

## Abstract

Nucleotide-binding oligomerization domain leucine rich repeat and pyrin domain-containing protein 3 (NLRP3) inflammasome is pivotal in maintaining intestinal homeostasis and sustaining enteric immune responses in the setting of inflammatory bowel diseases. Drugs acting as NLRP3 blockers could represent innovative strategies for treatment of bowel inflammation. This study was performed in rats with dinitrobenzenesulfonic acid (DNBS)-induced colitis, to investigate how the direct blockade of NLRP3 inflammasome with an irreversible inhibitor (INF39) compares with Ac-YVAD-cmk (YVAD, caspase-1 inhibitor) and anakinra (IL-1β receptor antagonist), acting downstream on NLRP3 signaling. Animals with DNBS-colitis received YVAD (3 mg/kg) or anakinra (100 mg/Kg) intraperitoneally, and INF39 (25 mg/kg) or dexamethasone (DEX, 1 mg/kg) orally for 6 days, starting on the same day of colitis induction. Under colitis, there was a body weight decrease, which was attenuated by YVAD, anakinra or INF39, but not DEX. All test drugs counteracted the increase in spleen weight. The colonic shortening and morphological colonic alterations associated with colitis were counteracted by INF39, anakinra and DEX, while YVAD was without effects. Tissue increments of myeloperoxidase, tumor necrosis factor and interleukin-1β were more effectively counteracted by INF39 and DEX, than YVAD and anakinra. These findings indicate that: (1) direct inhibition of NLRP3 inflammasome with INF39 is more effective than caspase-1 inhibition or IL-1β receptor blockade in reducing systemic and bowel inflammatory alterations; (2) direct NLRP3 inhibition can be a suitable strategy for treatment of bowel inflammation.

## Introduction

Inflammatory bowel diseases (IBDs), including Crohn’s disease and ulcerative colitis, are chronic relapsing disorders characterized by inflammation and tissue damage in the digestive tract (Neurath, 2014). Such diseases are associated with marked morbidity and have a remarkable negative impact on patients’ quality of life, which highlights the need for setting up novel anti-inflammatory therapeutic strategies (Blonski et al., 2011).

Recent studies have shown that the nucleotide-binding oligomerization domain, leucine-rich repeat and pyrin domain containing protein 3 (NLRP3) inflammasome cytosolic complex, besides acting as a key player in the maintenance of intestinal homeostasis, shapes innate immune responses against commensal bacteria. Indeed, NLRP3 over-activation during bowel inflammation is associated with a breakdown of enteric immune balance, suggesting an involvement of NLRP3 in the pathogenesis of bowel inflammation (Bauer et al., 2010; Kanneganti, 2017; Pellegrini et al., 2017b).

The activation of NLRP3 inflammasome requires two parallel and independent steps: transcription and oligomerization (Martín-Sanchez et al., 2016; Gaidt and Hornung, 2018; Mangan et al., 2018). The first step is regulated by innate immune signaling, mediated primarily by toll-like receptors (TLRs), myeloid differentiation primary response 88 (MyD88) and/or cytokine receptors, such as TNF receptor, which, in turn, activate pro-IL-1β and NLRP3 transcription *via* NF-κB activation. The second step results in NLRP3 inflammasome oligomerization, leading to caspase-1 activation and, in turn, IL-1β and IL-18 processing and release (Sutterwala et al., 2014).

Of note, clinical evidence has documented an increase in IL-1β release from colonic tissues and macrophages of IBD patients, these patterns being correlated with disease severity, thus suggesting IL-1β as a relevant pro-inflammatory cytokine involved in the pathophysiology of IBDs (Coccia et al., 2012). Given the involvement of the inflammasome pathway in the pathophysiology of intestinal inflammation, current research efforts are being focused on the potential therapeutic benefits resulting from the pharmacological modulation of NLRP3 inflammasome. In this respect, previous studies investigated the role of NLRP3 inflammasome in several experimental models of colitis, highlighting remarkable beneficial effects as a results of its pharmacological modulation (Pellegrini et al., 2017b). For instance, two recent studies showed that both the *in vivo* caspase-1 inhibition and the selective blockade of NLRP3 inflammasome complex significantly attenuated colonic inflammation in spontaneous colitis mice (Zhang et al., 2014; Perera et al., 2018). In addition, Stoffels et al. (2014) reported that anakinra reduced post-operative inflammation and ameliorated post-operative ileus in mice, thus suggesting that IL-1β receptor blockade exerts beneficial effects during intestinal inflammation (Stoffels et al., 2014). Moreover, in a recent paper, we observed that the *in vivo* direct irreversible inhibition of NLRP3 with INF39, a novel acrylate compound able to inhibit NLRP3 ATPase activity, exerts beneficial effects on bowel inflammation (Cocco et al., 2017).

These findings suggest that both upstream and downstream inhibition of NLRP3 inflammasome could represent suitable pharmacological approaches to the treatment of bowel inflammation. However, a direct comparative study on the efficacy of NLRP3 inflammasome signaling inhibitors in a pre-clinical model of bowel inflammation is lacking.

Based on this background, the present study was designed to investigate how the direct blockade of NLRP3 with INF39 in experimental colitis compares, in terms of efficacy, with other drugs acting downstream (caspase-1 inhibition or IL-1β receptor blockade) on NLRP3 signaling.

## Materials and Methods

### Animals

Experimental procedures were performed on male Sprague-Dawley rats, 200–250 g body weight. The animals received standard laboratory chow and tap water without any restriction and were housed, three in a cage, in temperature- controlled rooms on a 12-h light cycle at 22–24°C and 50–60% humidity. Animal care and handling were in accordance with the provisions of the European Community Council Directive 2010/63/UE, recognized and adopted by the Italian Government. All experimental procedures were approved by the Ethical Committee for Animal Experimentation of the University of Pisa and by the Italian Ministry of Health (authorization n° 674/2016-PR). In the present study, animal data have been presented according to the ARRIVE guidelines.

### Induction of Colitis and Drug Treatments

Colitis was induced in accordance with the method described previously (Antonioli et al., 2010). The subsequent experimental procedures were performed 6 days after DNBS administration to allow a full development of colonic inflammation. Rats were subjected to administration of the test drugs by intragastric or intraperitoneal (i.p.) route for 6 days, starting on the same day of DNBS injection. DNBS-untreated (controls) and DNBS-treated animals were treated as follows: INF39 (25 mg/kg/day, oral), Ac-YVAD-cmk (YVAD, caspase-1 inhibitor, 3 mg/Kg/day, i.p.), anakinra (IL-1β receptor antagonist, 100 mg/Kg/day i.p.) or DEX (active comparator, 1 mg/kg/day, oral). INF39 and DEX were suspended in olive oil and 1% methylcellulose, respectively. YVAD was dissolved in sterile DMSO, and further dilutions were made with sterile saline.

Subgroups of DNBS-untreated and DNBS-treated rats received drug vehicles to serve as controls. Body weight was assessed daily, starting from the onset of drug administrations. The doses of INF39 and DEX were selected on the basis of our previous study performed on the same rat model of colitis (Cocco et al., 2017).

The doses of YVAD and anakinra were selected on the basis of previous studies performed in rat models of diabetes induced by stress or in streptozotocin, respectively (Maslanik et al., 2013; Vallejo et al., 2014), and by preliminary experiments designed to test increasing doses of both compounds (YVAD, 0.75, 1.5, 3, and 6 mg/kg; anakinra, 25, 50, 100, and 200 mg/Kg) on colonic MPO levels in the model of DNBS-induced colitis. Macroscopic and histological scores were assessed on the whole colon, while biochemical assays were performed on colonic segments collected from an inflamed region adjacent and distal to the gross necrotic damage.

### Assessment of Colitis

Colonic tissues were scored for macroscopic and histological damage, as reported previously (Antonioli et al., 2010; Cocco et al., 2017). The criteria for macroscopic scoring of colonic damage were as follows: (1) presence of adhesions between colonic tissue and other organs (0, none; 1, minor; 2, major adhesions); (2) consistency of colonic fecal material (0, formed; 1, loose; 2, liquid stools); and (3) presence of ulceration (0, none; 1, hyperemia; 2, ulceration without hyperemia; 3, ulceration with inflammation at one side; 4, ≥ 2 sites of ulceration and inflammation; 5, major sites of damage; and 6, major sites of damage extending > 2 cm). The score was then increased by one unit for each millimeter of colonic wall thickness. Microscopic damage and inflammation were assessed by light microscopy on hematoxylin/eosin-stained histological sections obtained from whole gut specimens. The histological criteria included mucosal architecture loss (0 –3), cellular infiltrate (0 –3), muscle thickening (0 –3), crypt abscess (0, absent; 1, present), and goblet cell depletion (0, absent; 1, present). All parameters of macroscopic and histological damages were recorded and scored for each rat by two observers blinded to the treatment. At the time of experiment, the weight of spleen and the colonic length were also measured.

### Evaluation of Tissue Myeloperoxidase Levels

The evaluation of MPO levels, regarded as quantitative index for quantification of the degree of intestinal tissue infiltration by polymorphonuclear cells, was performed as described previously (Fornai et al., 2016). Colonic samples (30 mg) were put in 0.6 ml of ice-cold lysis buffer containing 200 mM NaCl, 5 mM EDTA, 10 mM Tris, 10% glycerine, 1 mM phenylmethylsulfonyl fluoride, 1 g/ml leupeptin and 28 g/ml aprotinin (pH 7.4) and homogenized on ice with a polytron homogenizer (QIAGEN, Milan, Italy). After centrifugation (2 times at 4°C for 15 min at 1,500*g*), the supernatant was diluted 1:5 and used for MPO concentration assessment by an enzyme-linked immunosorbent assay (ELISA) (Hycult Biotech, Uden, Netherlands). Data were expressed as nanograms of MPO per milligram of colonic tissue.

### Evaluation of Tissue TNF and IL-1β Levels

The evaluation of TNF and IL-1β levels in colonic tissues was performed by an ELISA kit (Abcam), as described previously by Pellegrini et al. (2017a). Briefly, colonic samples, were weighed and homogenized in 0.4 ml of PBS, pH 7.2/20 mg of tissue at 4°C, and centrifuged at 10,000*g* for 5 min. Supernatants were employed for the assay. The concentrations of TNF and IL-1β were expressed as picograms and nanograms per milligram of tissue, respectively.

### Drugs and Reagents

Dimethyl sulfoxide, DNBS, DEX, Ac-YVAD-cmk (caspase-1 inhibitor) and methylcellulose were purchased from Sigma-Aldrich (St. Louis, MO). The synthesis of INF39 was performed as previously reported (Cocco et al., 2014, 2016, 2017). Anakinra was purchased from Sobi, Swedish Orphan Biovitrum s.r.l. (Parma, Italy).

## Statistical Analysis

The results are presented as mean ± S.E.M. unless otherwise stated. Data have been reported also synoptically in Table 1 as percent changes against the values estimated in animals with colitis for the purpose of support to the discussion. The significance of differences was evaluated on raw data by one-way analysis of variance followed by *post hoc* analysis with Student-Newman-Keuls test. *P*-values < 0.05 were considered significantly different. All statistical procedures were performed by a commercial software (GraphPad Prism, version 7.0 from GraphPad Software Inc., San Diego, CA, United States).

**Table 1:** Synoptic presentation of percent changes of tissue-related inflammatory parameters in DNBS-rats treated with YVAD, anakinra, INF39 or DEX against values estimated in animals with colitis.

	Colon length	Macroscopic damage score	Microscopic damage score	MPO	TNF	IL-1β
YVAD	+ 7.6	−38.1	−37.7	−61.5	+18.5	−32.4
Anakinra	+ 20.1	−55.1	−50.9	−66.7	+ 4.5	−12.6
INF39	+ 22.1	−62.8	−67.8	−83.4	−50.5	−65.5
DEX	+ 18.4	−71.8	−73.5	−90.1	−57.4	−76.3

*DEX, Dexamethasone; DNBS, 2,4-dinitrobenzenesulfonic acid; TNF_,_ tumor necrosis factor; IL-lβ, interleukin-l beta; MPO:myeloperoxidase.*

## Results

### Body Weight, Spleen Weight and Colonic Length

The administration of INF39, YVAD, anakinra and DEX to animals treated with DNBS vehicle did not elicit any significant change in both systemic and tissue parameters (data not shown). On this basis, we adopted the animals treated with DNBS vehicle rats as control group for all the evaluations on the drugs under investigation, collectively designed as test drugs.

Six days after administration of the DNBS vehicle, control rats showed a weight gain of 19.6 ± 0.9 g, while rats treated with DNBS displayed a decrease of 36.6 ± 4.4 g in their body weight. In rats with colitis, treatment with YVAD, anakinra and INF39 significantly attenuated the body weight loss, while DEX promoted a further, although not significant, decrease in body weight. In this respect, it is widely recognized that steroid therapy is associated with several systemic adverse effects, including muscular atrophy, with consequent loss of body weight (De Cassan et al., 2012).

**FIGURE 1:**
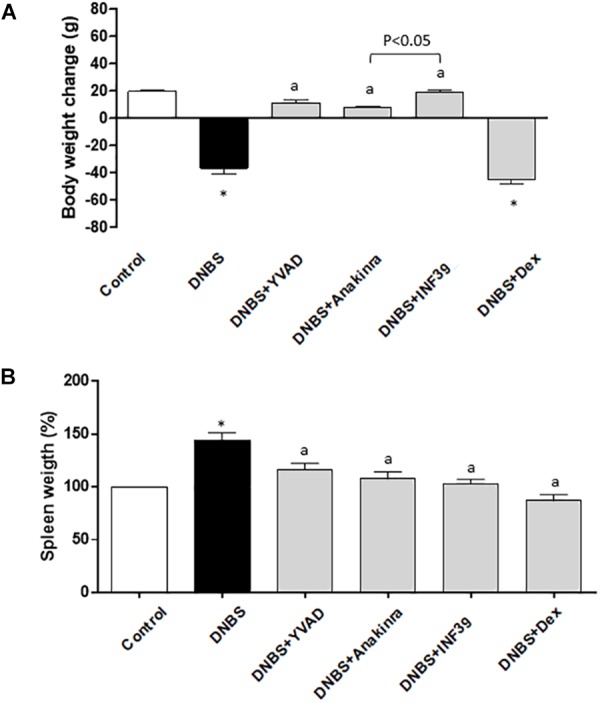
Effects of Ac-YVAD-cmk (YVAD, 3 mg/Kg), anakinra (100 mg/Kg), INF39 (25 mg/kg) or DEX (1 mg/kg) on body weight gain/loss **(A)** and spleen weight changes **(B)**, at day 6 after induction of colitis with DNBS. Each column represents the mean ± S.E.M. (*n* = 10). ^∗^*P* < 0.05, significant difference vs. control group; ^a^*P* < 0.05, significant difference vs. DNBS group.

Of note, INF39 was significantly more effective in blunting body weight loss, as compared with anakinra (Figure 1), suggesting that the upstream inhibition of NLRP3 activation is more effective than the downstream IL-1β receptor blockade.

Spleen weight was taken as an index of systemic inflammation (Chassaing et al., 2014) (Siegmund et al., 2001). Treatment with DNBS resulted in a significant increment of spleen weight (+44.3%), as compared with control animals. Such an increase was significantly counteracted to a similar extent by YVAD, anakinra and INF39 (Figure 1B).

**FIGURE 2:**
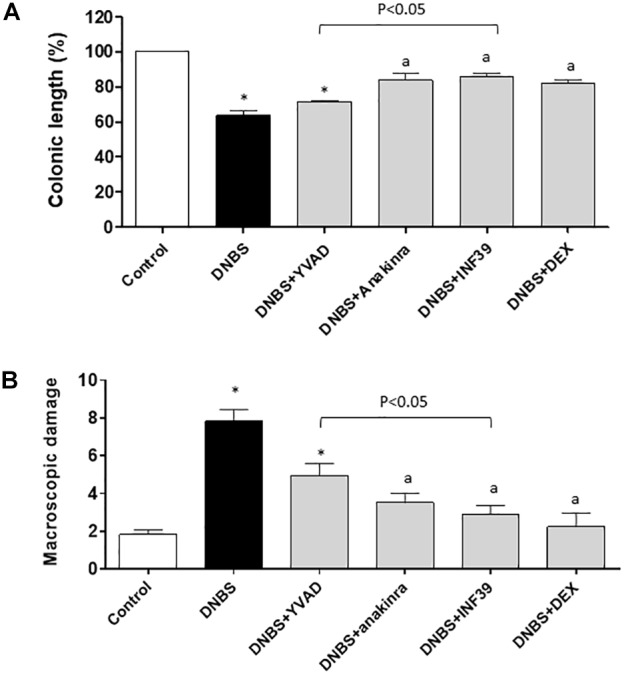
Colonic length changes **(A)** and macroscopic damage score **(B)** in rats under normal conditions or with DNBS-induced colitis, either alone or after treatment with Ac-YVAD-cmk (YVAD, 3 mg/Kg), anakinra (100 mg/Kg), INF39 (25 mg/kg) or DEX (1 mg/kg). Each column represents the mean ± S.E.M. (*n* = 10). ^∗^*P* < 0.05, significant difference vs. control group; ^a^*P* < 0.05, significant difference vs. DNBS group.

Six days after DNBS administration, inflamed rats were characterized by a shortening of colonic length (−37.2%), as compared with control animals. Treatment of inflamed rats with anakinra, DEX or INF39 attenuated significantly the decrease in colonic length, while YVAD did not exert significant effects; (Figure 2A and Table 1), suggesting that both the upstream and downstream inhibition of NLRP3 signaling with INF39 and anakinra, respectively, is more effective than caspase-1 inhibition in counteracting the decrease in colonic length.

### Macroscopic Damage

The administration of DNBS was associated with colonic thickening and ulcerations, with marked areas of transmural inflammation. In addition, adhesions and bowel dilations were detected, with a macroscopic damage accounting for 7.8 ± 0.6. In this setting, the macroscopic damage was reduced to a similar extent and significantly by anakinra, INF39 and DEX, while the effect of YVAD did not achieve statistical significance; anakinra and INF39 acted with similar efficacy (Figure 2B and Table 1).

### Microscopic Damage

Microscopic evaluation of colonic tissues revealed the presence of large areas of mucosal necrosis in animals treated with DNBS, as well as the destruction of glandular architecture.

**FIGURE 3:**
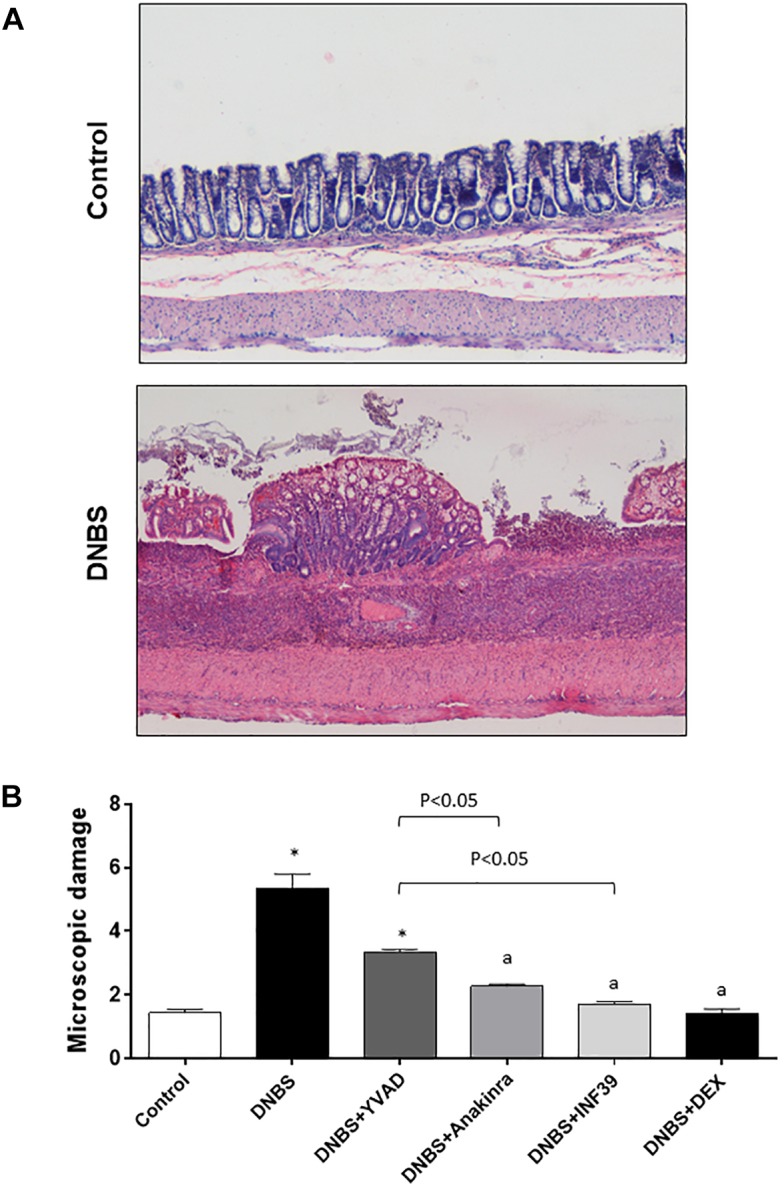
**(A)** Hematoxylin and eosin-stained sections of rat colon. Representative microscopic images refer to control rats or animals with colitis induced by DNBS. **(B)** Microscopic damage score estimated for colon in rats under normal conditions or with DNBS-induced colitis, either alone or after treatment with Ac-YVAD-cmk (YVAD, 3 mg/Kg), anakinra (100 mg/Kg), INF39 (25 mg/kg) or DEX (1 mg/kg). Each column represents the mean ± S.E.M. (*n* = 10). ^∗^*P* < 0.05, significant difference vs. control group; ^a^*P* < 0.05, significant difference vs. DNBS group.

The submucosa appeared thickened due to the presence of edema and inflammatory cell infiltration. The underlying muscular layer appeared also thickened and infiltrated with inflammatory cells following DNBS administration. The mucosa and submucosa surrounding the necrotic area displayed inflammation associated with marked cellular infiltration, as compared with tissues from control animals (Figure 3A).

In colonic tissues from inflamed rats, the microscopic score was significantly increased in comparison with control animals (5.3 ± 0.4 vs. 1.4 ± 0.1, respectively). When treated with anakinra, INF39 and DEX, animals with colitis displayed similar and significant reductions of the microscopic damage, while the effect of YVAD did not achieve the level of statistical significance (Figure 3B and Table 1).

### MPO Levels in Colonic Tissues

**FIGURE 4:**
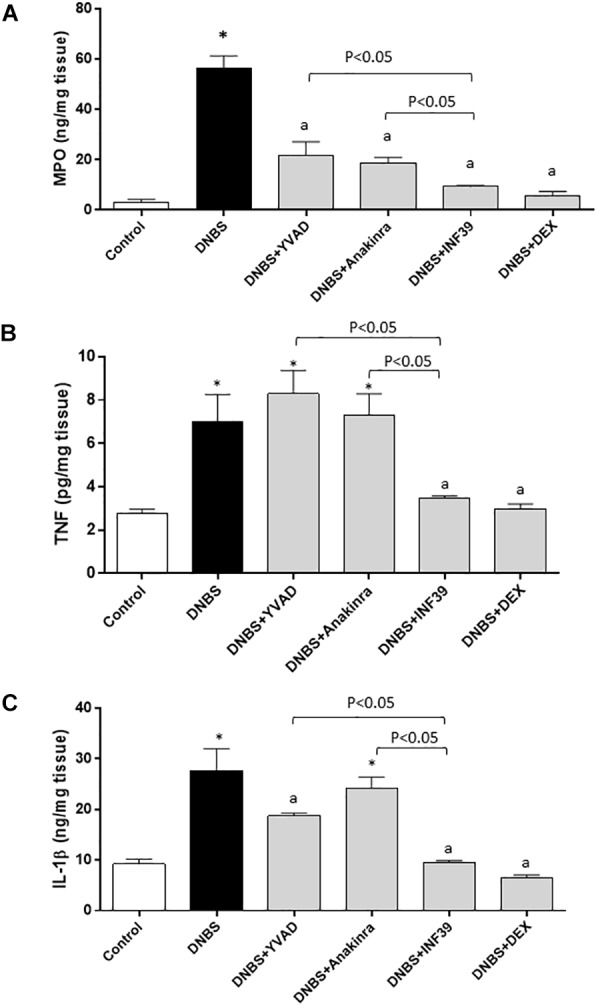
Myeloperoxidase (MPO) **(A)**, tumor necrosis factor (TNF) **(B)** and interleukin-1β (IL-1β) **(C)** levels in colonic tissues from control rats or animals with DNBS-induced colitis, either alone or after treatment with Ac-YVAD-cmk (YVAD, 3 mg/Kg), anakinra (100 mg/Kg), INF39 (25 mg/kg) or DEX (1 mg/kg). Each column represents the mean ± S.E.M. (*n* = 10). ^∗^*P* < 0.05, significant difference vs. control group; ^a^*P* < 0.05, significant difference vs. DNBS group.

Rats with DNBS-induced colitis showed a marked increase in colonic MPO levels (56.3 ± 4.9 ng/mg tissue), as compared with control animals (3.2 ± 1.1 ng/mg tissue). Treatment with all test drugs prevented significantly the increments of colonic MPO levels associated with DNBS administration, with INF39 being more effective than YVAD and anakinra (Figure 4A and Table 1).

### TNF Levels in Colonic Tissues

Colonic inflammation induced by DNBS was associated with a significant increase in tissue TNF levels (7.1 ± 1.2 pg/mg tissue), as compared with values obtained in control animals (2.7 ± 0.2 pg/mg tissue). Treatment with INF39 or DEX decreased significantly the concentration of this cytokine in colonic tissues, while YVAD and anakinra were without effects (Figure 4B and Table 1).

### IL-1β Levels in Colonic Tissues

Rats with colitis displayed a significant increase in colonic IL-1β levels (27.7 ± 4.2 ng/mg tissue), as compared with values estimated in control animals (9.2 ± 0.9 ng/mg tissue). Treatment with YVAD, INF39 and DEX was associated with a significant decrease in IL-1β levels; INF39 was more effective than YVAD. Anakinra was without effects (Figure 4C and Table 1).

## Discussion

The involvement of inflammasome pathways in the pathophysiology of intestinal inflammation is fostering research about the potential therapeutic benefits, in terms of anti-inflammatory activity, resulting from the pharmacological modulation of NLRP3 inflammasome. Nowadays, the majority of available studies have investigated the role of NLRP3 in several experimental models of colitis, highlighting remarkable beneficial effects associated with the pharmacological modulation of this enzymatic complex (Pellegrini et al., 2017b). In particular, the most investigated strategies, aimed at validating and developing novel pharmacological entities targeting NLRP3 signaling, have been: (i) inhibition of the activation of the transcription factor NF-κB; (ii) protection against mitochondrial damage; (iii) activation of the Keap-1/Nrf2 antioxidant pathway; (iv) inhibition of pro-caspase-1 cleavage through undetermined interactions with NLRP3 inflammasome; and (v) blockade of IL-1β receptor (Sutterwala et al., 2014; Cocco et al., 2017; Perera et al., 2017). However, at present, there is a lack of suitable drug candidates able to directly and selectively inhibit the NLRP3 ATPase activity. This represents an intriguing issue, since the development of drugs endowed with direct inhibitory effects on NLRP3 are expected to ensure a more efficient control of several NLRP3-dependent downstream signals, pivotally involved in the regulation of immune/inflammatory processes (Pellegrini et al., 2017b).

The above background was taken as a rationale for the development of INF39, an acrylate compound, able to block selectively and irreversibly the NLRP3 ATPase activity (Cocco et al., 2017). *In vitro* studies showed that INF39 counteracted significantly NLRP3 activation through a direct and irreversible interaction with the enzyme complex (Cocco et al., 2017). The impact of a direct NLRP3 blockade on bowel inflammation was then tested in a murine model of DNBS-induced colitis (Cocco et al., 2017). In this setting, INF39 exerted significant beneficial effects, alleviating both the systemic and tissue inflammatory outcomes of colitis, and showing also a satisfactory safety profile (Cocco et al., 2017).

In the present study, our specific purpose was to compare the efficacy of INF39 with other drugs, acting downstream on the NLRP3 signaling (i.e., caspase-1 inhibition or IL-1β receptor blockade), in an experimental model of bowel inflammation. To pursue this goal, we employed rats with DNBS-induced colitis, which match closely the patterns of Crohn’s disease in humans, and are characterized by body weight loss, diarrhea, ulceration and bleeding, depletion of goblet cells and formation of granulomas within the colonic wall (Goyal et al., 2014). The model of DNBS-induced colitis is a suitable tool for the investigation of the anti-inflammatory activity of novel drugs with potential therapeutic efficacy in human bowel inflammatory disorders (Esposito et al., 2010). The appropriateness of this model was previously validated by our research group. Indeed, we confirmed DEX was able to ameliorate systemic and tissue inflammatory parameters, as also previously described by Antonioli et al. (2010).

Overall, our results provide convincing evidence that the pharmacological modulation of the NLRP3 inflammasome pathway attenuates bowel inflammation in DNBS-induced colitis, and that direct NLRP3 inhibition by INF39 is more effective than caspase-1 inhibition by YVAD or IL-1β receptor blockade by anakinra in controlling several parameters associated with intestinal inflammation. When considering the systemic indexes of inflammation, treatment with INF39, YVAD and anakinra counteracted the body weight loss and the increment of spleen weight in DNBS-rats. In addition, the inhibitors of NLRP3 signaling did not exert detrimental effects on body weight, at variance with DEX, which, being a systemically acting glucocorticoid derivative, is known to be associated with a variety of adverse effects, including muscular atrophy (De Cassan et al., 2012). When analyzing colonic length and bowel morphological parameters, INF39 and anakinra counteracted significantly the colonic shortening and improved both the macroscopic and histological features of colitis, as compared with YVAD, thus suggesting that the direct inhibition of NLRP3 or IL-1β receptor blockade can exert significant beneficial effects on tissue parameters related to inflammation than caspase-1 inhibition. Of note, when considering body weight and colonic length, we observed heterogeneous effects of the drugs under investigation. In this respect, it is noteworthy that body weight and colonic length in animals with colitis, despite being widely employed, are colonic coarse parameters, which can be associated also with high variability, as compared with the assessment of colonic microscopic damage or colonic MPO, TNF and IL-1β levels (see below). This is a relevant point since, when testing the effects of drugs on coarse parameters the net effect of individual drugs could be less evident. As a consequence, this circumstance might explain why anakinra was less effective than INF39 in attenuating body weight, or why YVAD did not exert a significant effect on colonic length.

Taken together, the present findings are in line with recent studies showing that treatment with MCC950, a potent and highly specific small molecule inhibitor of NLRP3 inflammasome counteracted bowel inflammation in a spontaneous colitis murine model (Winnie mice), and, that anakinra reduced post-operative inflammation and ameliorated post-operative ileus in mice (Stoffels et al., 2014; Perera et al., 2018). In addition, two recent studies showed that a synthetic benzimidazole compound and fumigaclavine C, a fungal metabolite exerted anti-inflammatory effects in mice with colitis induced by dextran sulfate sodium (DSS), through the inhibition of caspase-1 activation (Liu et al., 2013; Guo et al., 2015). However, although both compounds were able to inhibit caspase-1 activation, they influenced also other intracellular pathways, including MAPK, STAT1 and NF-κB signaling. Therefore, their anti-inflammatory effects on colitis may not result merely from the inhibition of caspase-1.

A variety of inflammatory mediators have been shown to take a significant part in the pathogenesis of bowel inflammation (Neurath, 2014). In particular, it is recognized that TNF plays a pivotal role, both in humans and experimental colitis models in the production of chemoattractants for neutrophils and their activation. Activated neutrophils infiltrate the mucosa and submucosa, and contribute to intestinal injury through a number of mechanisms (Neurath, 2014). This body of knowledge explains why anti-cytokine therapies based on TNF-specific blocking agents represent an important cornerstone of medical therapy in both Crohn’s disease and ulcerative colitis (Danese and Fiocchi, 2011; Baumgart and Sandborn, 2012). Taking this rationale into account, we examined the effects of all test drugs on the colonic levels of TNF and MPO (a quantitative index reflecting the degree of colonic infiltration by polymorphonuclear cells) in DNBS-rats. Interestingly, our results showed that INF39, but not YVAD and anakinra, counteracted significantly the increments of TNF, and that INF39 blunted the increased MPO levels with greater efficacy than the caspase-1 inhibitor and IL-1β receptor antagonist in rats with colitis, thus corroborating the concept that the direct blockade of NLRP3 could represent a better pharmacological strategy for treatment of bowel inflammation, than caspase-1 inhibition and IL-1β receptor blockade (Pellegrini et al., 2017b).

The above results are in line with a recent study showing that treatment of animals with spontaneous chronic colitis (Winnie mice) with a specific NLRP3 inhibitor (MCC950) counteracted bowel inflammation, improving both the macroscopic and histological features of colitis and reducing significantly the release of pro-inflammatory cytokines, including TNF levels (Perera et al., 2018).

Of note, the peculiar effect of INF39 in downregulating TNF tissue levels depends very likely on its ability to modulate TNF gene expression. Indeed, as observed in our previous study, the exposure of bone marrow-derived macrophages, stimulated with lipopolysaccharide, to INF39 was followed by a significant decrease in TNF gene expression, as compared with INF39-untreated cells (Cocco et al., 2017).

Several lines of pre-clinical and clinical evidence point out a key role of IL-1β in the pathophysiology of IBDs (Neurath, 2014). Indeed, colonic tissues and *lamina propria* macrophages from IBD patients showed an increased IL-1β secretion, this activity being well correlated with disease severity (Gustot et al., 2005; Coccia et al., 2012). A significant activation of NLRP3 inflammasome, with a consequent massive release of IL-1β, has been detected previously in monocytes infiltrating the lamina propria and M1 pro-inflammatory macrophages isolated from intestinal specimens of IBD patients (Lissner et al., 2015). For this reason in the present study, sets of experiments were devoted to evaluate the efficacy of test drugs in counteracting IL-1β release. In this setting, treatment with INF39 was more effective than anakinra and YVAD in counteracting the increase in IL-1β levels in colonic tissues from DNBS-rats. Anakinra, being a receptor blocker, was not expected indeed to modify IL-1β release. Furthermore, even though anakinra is currently employed for treatment of some immune-mediated inflammatory diseases (i.e., rheumatoid arthritis, ankylosing spondylitis and gout) (Dinarello et al., 2012), scarce beneficial effects of this drug were observed in patients with IBDs, thus indicating the selective blockade of IL-1β receptor as a loosing strategy for the management of such diseases (Carter et al., 2003; Hügle et al., 2017). Indeed, although several pre-clinical studies in animal models of colitis showed that the inhibition of IL-1β decreased tissue inflammation and necrosis, others found few or no beneficial effects following IL-1β inhibition (de Mooij et al., 2017). In addition, in a previous study, Carter et al. (2003) reported that the administration of anakinra to patients with Crohn’s disease was associated with a worsening of clinical conditions, including fever, increased diarrhea and abdominal pain.

When considering the caspase-1 blocker YVAD, recent studies have shown that, besides caspase-1, a non-canonical NLRP3 inflammasome activation, which depends on caspase-11, plays a significant role also both in the maintenance of intestinal immune homeostasis and in sustaining the pathophysiological events that underlie bowel inflammation (Kayagaki et al., 2011; Vigano and Mortellaro, 2013). In particular, caspase-11 overactivation during bowel inflammation contributes to the massive release of IL-1β through activation of the NLRP3-ASC-caspase-1 pathway, and the inhibition of both canonical and non-canonical caspase-11-dependent NLRP3 activation has been found to exert anti-inflammatory effects on colitis in mice (Márquez-Flores et al., 2016). Other studies on murine bone marrow-derived dendritic cells (BMDCs) have shown that, besides canonical and non-canonical caspase-11-dependent NLRP3 activation, a caspase-8-dependent NLRP3 inflammasome activation can be called into play also to promote the processing and release of IL-1β. Indeed, IL-1β processing and caspase-8 activation were not evident in *Nlrp3^−/−^*or *Asc^−/−^* BMDCs, thus indicating that caspase-8 can act as a direct IL-1β-converting enzyme (Antonopoulos et al., 2015;Chung et al., 2016). Moreover, Gringhuis et al. (2012) showed that the release of IL-1β from dendritic cells, stimulated with a fungal infection, occurred independently of caspase-1, but required an association of the inflammasome protein ASC with caspase-8. Based on these observations, it is conceivable that, in our experiments, INF39, through the specific inhibition of NLRP3 ATPase activity, ensured a blockade of both canonical and non-canonical caspase-8- and caspase-11-dependent NLRP3 activation, thus leading to a more effective blockade of IL-1β release, as compared with YVAD. Of interest, our results are consistent with the findings of a recent study, showing that the direct and selective blockade of inflammasome with MCC950, the most specific and well characterized NLRP3 inhibitor available so far, attenuated colonic inflammation in mice with spontaneous colitis, likely through the inhibition of both canonical and non-canonical NLRP3 activation (Perera et al., 2018). However, the possible role and respective significance of non-canonical caspase-8- and caspase-11-dependent NLRP3 activation in the pathophysiology of bowel inflammation requires confirmation by means of specific experimental approaches. In addition, further studies are needed to evaluate the effects of *in vivo* selective blockade of non-canonical caspase-8- and caspase-11-dependent NLRP3 activation in animal models of colitis.

Of note, given the pivotal role of NLRP3 in regulating the integrity of intestinal homeostasis, the possibility that its pharmacological blockade during bowel inflammation could interfere with healing processes should be taken into account. In this respect, several lines of evidence have shown that, in the early acute phases of inflammation, NLRP3 inflammasome activation contributes to tissue repair and maintenance of epithelial barrier integrity, while in the later chronic phase of colitis, the overactivation of NLRP3 promotes the differentiation of T cells into effector Th1 and Th17 phenotypes, which contribute to sustain the inflammatory response. In line with this concept, the pharmacological inhibition of NLRP3 inflammasome signaling has been shown to exert beneficial effects in several experimental models of colitis (Pellegrini et al., 2017b). However, the possibility that long-term treatments with NLRP3 inhibitors could interfere with mucosal healing cannot be ruled out and deserves further investigations.

## Conclusion

**FIGURE 5:**
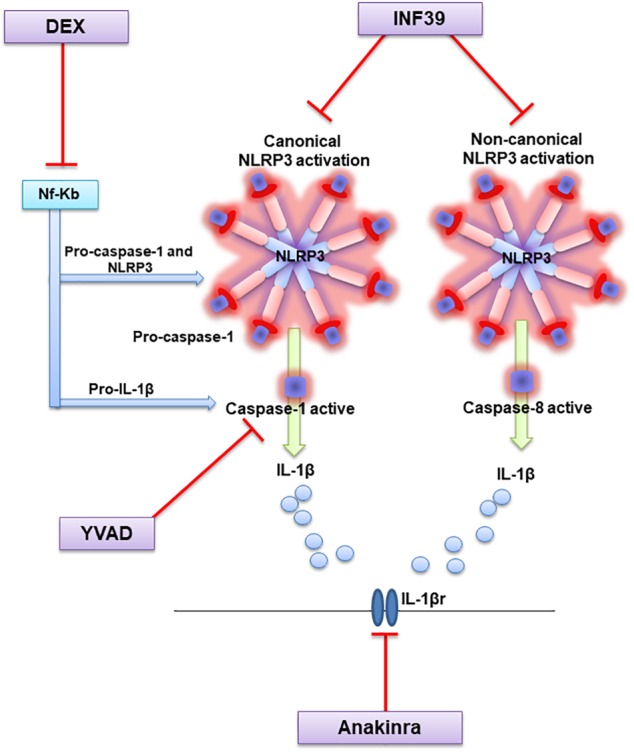
Diagram showing the molecular mechanisms through which test drugs may inhibit NLRP3 signaling and counteract intestinal inflammation.

In conclusion, our results expand current knowledge on the beneficial effects arising from the pharmacological modulation of NLRP3 inflammasome in experimental colitis (Pellegrini et al., 2017b), suggesting that the direct and irreversible inhibition of NLRP3 inflammasome complex could represent a more viable approach to the medical management of bowel inflammation than IL-1β receptor blockade or caspase-1 inhibition (see Figure 5). In keeping with this perspective, INF39 might represent a lead compound for the identification of novel NLRP3 inhibitors, characterized by high degrees of efficacy and concomitant favorable safety profiles. Therefore, the present observations might pave the way to the design and clinical development of novel NLRP3 selective inhibitors for the therapeutic management of patients with IBDs.

## Author Contributions

CP, LA, MB, CB, and MF participated in research design. MF, LA, RC, CP, VD’A, LB, and FF conducted the experiments. MB, GN, FF, SG, EM, and MG contributed new reagents or analytic tools. LA, CP, and RC performed the data analysis. CP, MF, RC, LA, and CB wrote or contributed to the writing of the manuscript.

## Conflict of Interest Statement

The authors declare that the research was conducted in the absence of any commercial or financial relationships that could be construed as a potential conflict of interest.

## Abbreviations

DEX: dexamethasone
DMSO: dimethyl sulfoxide
DNBS: 2,4-dinitrobenzenesulfonic acid
IL-1β: interleukin-1beta
MPO: myeloperoxidase
NF-κB: nuclear factor-κB
NLRP3: nucleotide-binding oligomerization domain leucine rich repeat and pyrin domain-containing protein 3
TNF: tumor necrosis factor.
